# New Developments at *EHP*

**DOI:** 10.1289/ehp.1509968

**Published:** 2015-04-01

**Authors:** Jane C. Schroeder, Susan M. Booker

**Affiliations:** 1 Interim Editor-in-Chief and; 2 News Editor, *Environmental Health Perspectives*, National Institute of Environmental Health Sciences, Research Triangle Park, North Carolina, USA

As many of you may recall, Hugh A. Tilson retired as Editor-in-Chief of *EHP* in August 2014. Historically, the Editor-in-Chief of *EHP* had two major functions: providing scientific and strategic vision, and managing day-to-day office operations. This dual role derived from *EHP*’s unique position as a government publication staffed by federal personnel, in which the head of the journal was also the head of an administrative branch. But the dual role is outdated and has not allowed the journal to grow in a way that best serves the field of environmental health sciences.

Managing operations to ensure manuscripts are processed and published in a timely fashion is a full-time job all by itself. Therefore, *EHP* has developed a new position to manage many of the operational duties once handled by the Editor-in-Chief. We are pleased to announce that Shaun Halloran has been hired as *EHP* ’s inaugural Operations Manager. Mr. Halloran was formerly Manager of Journals Production and Senior Manager of Production for the American Society of Civil Engineers, where he was responsible for more than 35 journals. As Operations Manager, Mr. Halloran serves as deputy to the Editor-in-Chief, overseeing manuscript production to ensure efficient and timely publication of the journal. *EHP* staff are excited about working with Shaun and bringing his experience to bear on journal operations.

Now that the Operations Manager position has been filled, *EHP* is seeking a new Editor-in-Chief. The job responsibilities for this position have been updated: the Editor-in-Chief will still function as the chief executive for the journal, but he or she will focus primarily on bringing a strategic vision to and managing all scientific aspects of the journal to ensure that *EHP* continues to publish articles of the highest quality.

The new Editor-in-Chief will need to be an internationally recognized leader in the environmental health sciences community and should have substantial experience in reviewing manuscripts and serving on editorial boards. The position will be posted on the USAJOBS website 30 March–17 April 2015 and will be filled within the next few months.

We are confident that filling these positions at *EHP* will help reduce our current backlog of manuscripts. We have made other recent changes that will help even more. Specifically, we have revised our process for final review of manuscripts, which will decrease the time from provisional acceptance to publication. Additionally, we have obtained a limited accessibility accommodation that will facilitate meeting our requirement, under Section 508 of the Rehabilitation Act, to ensure that all our electronic content is accessible to people with disabilities. As a federally funded publication, *EHP* is fully committed to 508 accessibility, yet producing an accessible document from an author’s manuscript can be surprisingly difficult and time-consuming, depending on the complexity of the paper. The result has been a significant strain on *EHP* ’s resources. With the new accommodation, *EHP* staff have developed an efficient strategy for providing accessible documents to readers who need them. We expect this to expedite publication.

We understand the frustration some authors have experienced with the delay in publication of their papers. We believe the steps we have taken will help alleviate this frustration. By hiring an Operations Manager, we expect to improve the efficiency of our production processes. We expect additional improvement with our new streamlined policies for final review of papers and meeting readers’ accessibility needs.

The editors and staff at *EHP* want to thank everyone, especially our authors and Associate Editors, for their ongoing support for the journal. As we continue making strides in addressing problematic issues, we look forward to your continued support.

