# Erratum: New Developments at *EHP*

**DOI:** 10.1289/ehp.123-A147

**Published:** 2015-06-01

Schroeder JC, Booker SM. 2015. New developments at *EHP*. Environ Health Perspect 123(4):A78; http://dx.doi.org/10.1289/ehp.1509968

Published online 1 April 2015

In the original version of this editorial Shaun Halloran’s previous employer was incorrectly stated as the American Society of Chemical Engineers. Mr. Halloran was employed by the American Society of Civil Engineers before coming to *EHP*.

*EHP* regrets the error.

